# RETRACTION: The Wound Adjuncts Effect of Closed Incision Negative Pressure Wound Therapy on Stopping Groin Surgical Site Wound Infection in Arterial Surgery: A Meta‐Analysis

**DOI:** 10.1111/iwj.70225

**Published:** 2025-02-11

## Abstract

**RETRACTION**: HongJ. , XieL. , FanL. , and HuangH. , “The Wound Adjuncts Effect of Closed Incision Negative Pressure Wound Therapy on Stopping Groin Surgical Site Wound Infection in Arterial Surgery: A Meta‐Analysis,” International Wound Journal 20, no. 7 (2023): 2726–2734, 10.1111/iwj.14146.36977282 PMC10410315

The above article, published online on 28 March 2023, in Wiley Online Library (http://onlinelibrary.wiley.com/), has been retracted by agreement between the journal Editor in Chief, Professor Keith Harding; and John Wiley & Sons Ltd. Following an investigation by the publisher, all parties have concluded that this article was accepted solely on the basis of a compromised peer review process. The editors have therefore decided to retract the article. The authors did not respond to our notice regarding the retraction.

**RETRACTION**: J. Hong , L. Xie , L. Fan , and H. Huang , “The Wound Adjuncts Effect of Closed Incision Negative Pressure Wound Therapy on Stopping Groin Surgical Site Wound Infection in Arterial Surgery: A Meta‐Analysis,” International Wound Journal 20, no. 7 (2023): 2726–2734, 10.1111/iwj.14146.36977282 PMC10410315

The above article, published online on 28 March 2023, in Wiley Online Library (http://onlinelibrary.wiley.com/), has been retracted by agreement between the journal Editor in Chief, Professor Keith Harding; and John Wiley & Sons Ltd. Following an investigation by the publisher, all parties have concluded that this article was accepted solely on the basis of a compromised peer review process. The editors have therefore decided to retract the article. The authors did not respond to our notice regarding the retraction.

